# Enabling robustness to failure with modular field robots

**DOI:** 10.3389/frobt.2024.1225297

**Published:** 2024-03-13

**Authors:** Troy Cordie, Jonathan Roberts, Matthew Dunbabin, Ross Dungavell, Tirthankar Bandyopadhyay

**Affiliations:** ^1^ CSIRO Robotics, Data61, Pullenvale, QLD, Australia; ^2^ Faculty of Engineering, Queensland University of Technology, QLD, Brisbane, Australia

**Keywords:** field robots, cellular and modular robots, uncrewed autonomous vehicles, space rovers, robot morphology, field robots

## Abstract

Actuator failure on a remotely deployed robot results in decreased efficiency or even renders it inoperable. Robustness to these failures will become critical as robots are required to be more independent and operate out of the range of repair. To address these challenges, we present two approaches based on modular robotic architecture to improve robustness to actuator failure of both fixed-configuration robots and modular reconfigurable robots. Our work uses modular reconfigurable robots capable of modifying their style of locomotion and changing their designed morphology through ejecting modules. This framework improved the distance travelled and decreased the effort to move through the environment of simulated and physical robots. When the deployed robot was allowed to change its locomotion style, it showed improved robustness to actuator failure when compared to a robot with a fixed controller. Furthermore, a robot capable of changing its locomotion and design morphology statistically outlasted both tests with a fixed morphology. Testing was carried out using a gazebo simulation and validated in multiple tests in the field. We show for the first time that ejecting modular failed components can improve the overall mission length.

## 1 Introduction

Robotic platforms deployed for remote operations can be left with no capacity for repair or replacement of parts. This isolation means that all problems encountered need to be overcome to keep the mission alive. Examples of remote operations include the Mars rover missions or operations on Earth where failure would leave the platform inaccessible. Problems faced while on these remote missions may include actuator failure or becoming bogged in the terrain. Previous missions to Mars were able to continue using compromised control strategies such as dragging a wheel. Missions have also ended when attempts to free a platform have been unavailable. This paper proposes the use of modular robotic architecture to increase the robustness of the deployed robots. The proposed framework has two approaches to improving the robustness of remotely deployed robots. Our first approach allows a robot to shift its instantaneous center of rotations (ICR) and change the style of locomotion, enabling continued operation with failed actuators, referred to as locomotion reconfigurability (LR). The second approach sees the robot eject the failed modules, allowing the platform to continue operations after becoming immobile through actuator failure or becoming stuck in the environment, referred to as design reconfigurability (DR).

**FIGURE 1:**
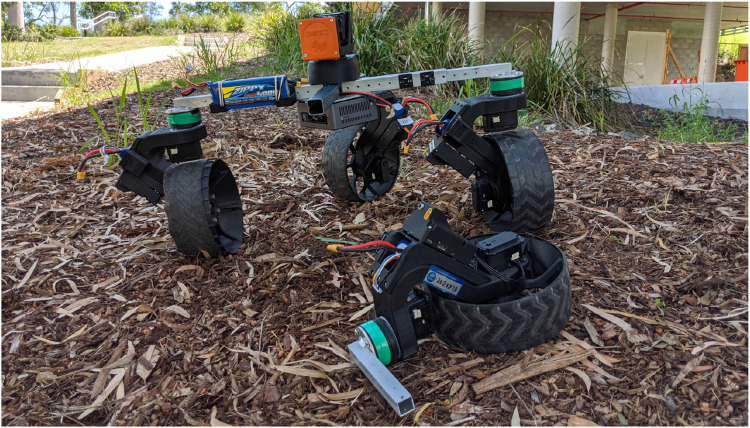
NeRobot modular robot system (NMRS) ejecting a failed NeWheel module.

The NeRobot modular robot system (NMRS) (Figure 1) serves as a testbed for developing these above-mentioned behaviors. In brief, the reconfigurable controller facilitates changing the locomotion style, while the modular design facilitates ejecting a failed module. The simulated NeRobot facilitated repeated testing of the proposed framework, measuring the distance travelled by the robot and the effort to move the platform. The framework was then implemented on the NMRS to validate the simulated results in the real world.

Results showed that the two proposed approaches improved robustness to actuator failure, increased the distance travelled, and decreased the effort of the platform to move. Of the two approaches, the ability to eject a module had the most impact on the distance travelled. The greatest improvement in the distance travelled occurred when adaptation using locomotion changes was exhausted before ejecting modules. These results show that robots deployed in remote locations, such as other planets, would benefit from modular architecture that increases the adaptability.

The contribution of this work is the validation of the robustness framework first described in the position paper presented at the International Astronautical Congress (Cordie et al., 2019a). This validation includes simulated deployments of robots to failure paired with field experiments in two environments and analysis of the resulting data. This analysis shows that both the simulated and physical robots saw a decrease in robot effort when using the proposed approach. Insights from the data provide guidance on when to eject failed modules to save energy or retain them to cover more distance.

## 2 Background

After the Russian Lunokhod-1 rover landed on the Moon in 1971 (Kassel, 1971), humans have continued to send rovers to extraterrestrial bodies. Once a rover is launched, they are beyond the reach of maintenance. The rovers survive unforeseen obstacles and component failure through their ability to adapt. Remotely deployed rovers operate until their actuators, sensors, or batteries decay, ending the mission. The Mars rovers Spirit and Opportunity exceeded their goal life span before succumbing to the limitations of their actuators. During operation, both rovers suffered actuator failure, resulting in altered control strategies (Townsend et al., 2014). Both rovers drove backward during their deployment to reduce the impact of these failures: Spirit to overcome a misaligned wheel, while Opportunity was reducing the load on a failing actuator (Showstack, 2010). Spirit operated with approximately 17% less efficiency until it became bogged down in 2009, operating as a static science platform until losing contact in 2010.

A class of robots addressing some of the issues faced by Spirit and Opportunity are the wheel-legged mobile robots (WLMRs). Designs vary significantly within this field. Individual robots differ in configuration and degrees of limb articulation, as seen in the kinematic models developed by Alamdari and Krovi (2016), Alamdari and Krovi (2014), Alamdari et al. (2013), and Sreenivasan and Wilcox (1994), as well as the generic models, such as those developed by Kelly and Seegmiller (2015). WLMRs can change their footprint, body clearance, and pose, as seen in existing platforms, including the All-Terrain Hex-Limbed Extra-Terrestrial Explorer (ATHLETE) (Wilcox et al., 2007; Howe et al., 2016), the Mars Analog Multi-Mode Traverse Hybrid (MAMMOTH) (Reid et al., 2016; Reid et al., 2014), the Scarab Lunar Drilling Rover (Bartlett et al., 2008), and the wheeled actively articulated vehicle (WAAV) (Sreenivasan et al., 1994; Sreenivasan and Waldron, 1996). Through reconfiguring its body, a WLMR could have overcome the misaligned wheel suffered by Opportunity. However, the noted ability of WLMRs to walk in the rough terrain (Klamt and Behnke, 2017) may have helped prevent Spirit from becoming bogged. However, this additional reconfigurability requires additional motors, sensors, and locking mechanisms, which, in turn, add to the mass, energy consumption, and system complexity (Machado and Silva, 2006). The additional mass and energy consumption are important factors for both transportation and deployment (Wilcox et al., 2007).

All of the WLMRs mentioned above would suffer the same fate as Spirit if a wheel became stuck. In this work, we have implemented and tested the work proposed in the paper by Cordie et al. (2019a), allowing a robot to reconfigure and adapt to failure. This adaptation combines the reconfigurability of the WLMRs with the noted ability of modular reconfigurable robots (MRRs) to degrade gracefully (Yim et al., 2007; Murata and Kurokawa, 2007). The simplest form of MRR is described by Murphy (2000) as a mother robot with a deployable daughter. An example of this type of robot is the 2020 Mars rover expedition, which plans to launch a daughter helicopter from the main rover (Moreno and Regina, 2018). A noted capability of MRRs is the ability to produce configurations specialized for different tasks from the same set of components (JIE et al., 2009; Salemi et al., 2006). If mother–daughter robots are the simplest modular robots, the modular, self-reconfigurable robots (MSRRs) represent the cutting edge of modular robotics. Typically consisting of homogeneous modules, systems such as SuperBot (Barrios et al., 2016; Chen et al., 2016) and SMORES-EP (Jing et al., 2016; Davey et al., 2012) illustrate the possibilities of MSRRs. This style of homogenous interconnecting modules is limited in the size of structures they can assemble by the actuator torque and the strength of the connection between the modules (Jing et al., 2016). For example, a SMORES-EP module is capable of actuating no more than 3.1 SMORES-EP modules when cantilevered, thus restricting the size and the maximum number of units in the robot. Platforms such as SnapBot (Kim et al., 2017; Ha et al., 2018; Ning et al., 2019) and Snake Monster (Kalouche et al., 2015) reduce the number of modules chained together by connecting modular actuators to a purpose-designed torso. However, the torso on both SnapBot and Snake Monster represents a single point of failure as it provides all communication and power to the limbs.

A recent review on robot failure by Liu et al. (2023) showed that most of the research studies focus on operational failures decoupled from system failures. Pan et al. (2022) looked at failures in task and motion and adapted their approaches based on the actions that failed during task execution. However, the hardware failures are not explicitly captured or triggered. Our work specifically focuses on hardware failures and provides a mechanism to mitigate the functional effect of such failures.

On the adaptive modeling perspective, many recent works have focused on Bayesian inference (Hammond et al., 2019), adapting the underlying world transition models (Pan et al., 2022) and learning the task feasibility model to deal with uncertainties during execution (Noseworthy et al., 2021). In terms of application, most of the focus has been on systems recovery via resolving programmatic deadlocks in the service robots (Das et al., 2021); Hammond et al., 2019) or, in the real world, mostly on grasping and bin-picking problems. Little work has been done on navigation in challenging and remote terrains, where manual intervention and robot repair are often not feasible. Our work shows the importance of varying the locomotion controller or the morphology of the robot in such scenarios.

This work incorporates the platform reconfigurability of wheel-legged robots with the ability to abandon faulty modules seen in modular robotics. Simulations and robotic demonstrations of this functionality use the NMRS. The NMRS contains homogeneous two degrees of freedom (DOF) modular wheels, or NeWheels, capable of independent or collaborative operation, combining one or more NeWheels with a dumb body using clamps and adaptors (Cordie et al., 2016 and Cordie et al., 2019b). The basis of the strategy explored was proposed by Cordie et al. (2019a); it involves internally reconfiguring the robot’s controller or locomotion reconfigurability and ejecting failed modules to adapt to failure.

## 3 Methods

A deployed reconfigurable modular wheeled robot facing actuator failure out of the range of repair could eject the defective module, performing DR. Ejecting a module would allow the robot to continue as an n-1 wheeled platform. However, this approach may reduce the capability of the robot and bring it closer to eventual failure. Modifying the control strategy through LR can allow a robot to continue operating without capability loss. Retaining modules is the priority until it represents a risk to completing the mission. We propose a two-step strategy for when a robot is faced with actuator failure.

• Step 1) Locomotion reconfigurability (LR); retain hardware and adapt to the failure through changes in the robot’s controller.
• Step 2) Design reconfigurability (DR); eject a failed module and adapt the controller based on the reconfigured robot.

**FIGURE 2:**
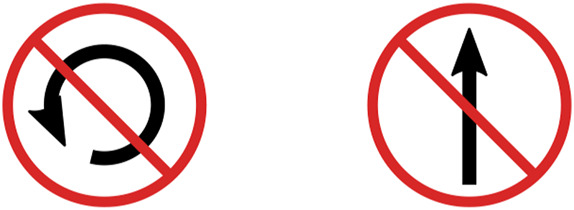
Icons indicating failure of an actuator from left to right: failure of a steering actuator and failure of the drive actuator.

After the implementation of step two, the cycle starts again at step one. Figure 5 depicts part of this cycle of a robot adapting from four modules to three and suffering subsequent failures. The potential failure modes are the failure of the drive actuator, the failure of a steering actuator, and the combined failure of the steering and drive actuators in a single module, referred to as “module failure”. These icons seen in Figure 2 will be used in plots and figures to indicate either the failed steering or the failed drive actuators.

### 3.1 NeRobot model

**FIGURE 3:**
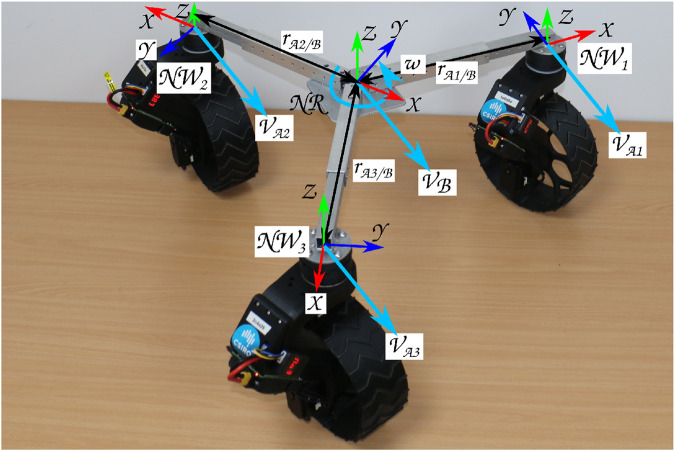
$C_{a_{3}}$ platform configured with the ICR at the center of three wheels. The combined velocity of the three connected wheels produces the desired linear and angular velocities of the body.

The NeRobot modular robot system, first detailed by Cordie et al. (2016) and Cordie et al. (2019b), allows fast reconfigurability before or during deployment. The robot model (Figure 3) is the link between the robot and the controller, where the robot is either a physical robot or a simulated version. The physical and simulated robots both consist of the same core components. The NeRobot model (NR 2) is composed of one or more NeWheels *NW*. Each *NW* includes the position of the hip joint *β*, the angular velocity of the wheels joint $\dot{\phi }$, and its pose [*x*,*y*]^*T*^ (NR 1).

$$NW={\left[\begin{array}{cccc} \beta & \dot{\phi } & x & y \end{array}\right]}^{T},$$

$$NR=\left[\begin{array}{ccccc} \beta_{1} & \beta_{2} & \beta_{3} & \ldots & \beta_{n}\\ \dot{\phi_{1}} & \dot{\phi_{2}} & \dot{\phi_{3}} & \ldots & \dot{\phi_{n}}\\ x_{1} & x_{2} & x_{3} & \ldots & x_{n}\\ y_{1} & y_{2} & y_{3} & \ldots & y_{n} \end{array}\right].$$

Values for *β* and $\dot{\phi }$ are received as feedback from each NeWheel to the controller. The parameter server provides values of the [*x*,*y*]^*T*^ set by the robot configuration file or updated during operation. The configuration file describes the robot at the starting time in a human-readable format, either describing the assembled configuration of a physical robot or using it to generate a simulated robot. The configuration file defines the number of NeWheel modules and their pose relative to the robot’s center of rotation. Additionally, the initial positions of the robot, joints, and sensors are defined.

### 3.2 Controller design

**FIGURE 4:**
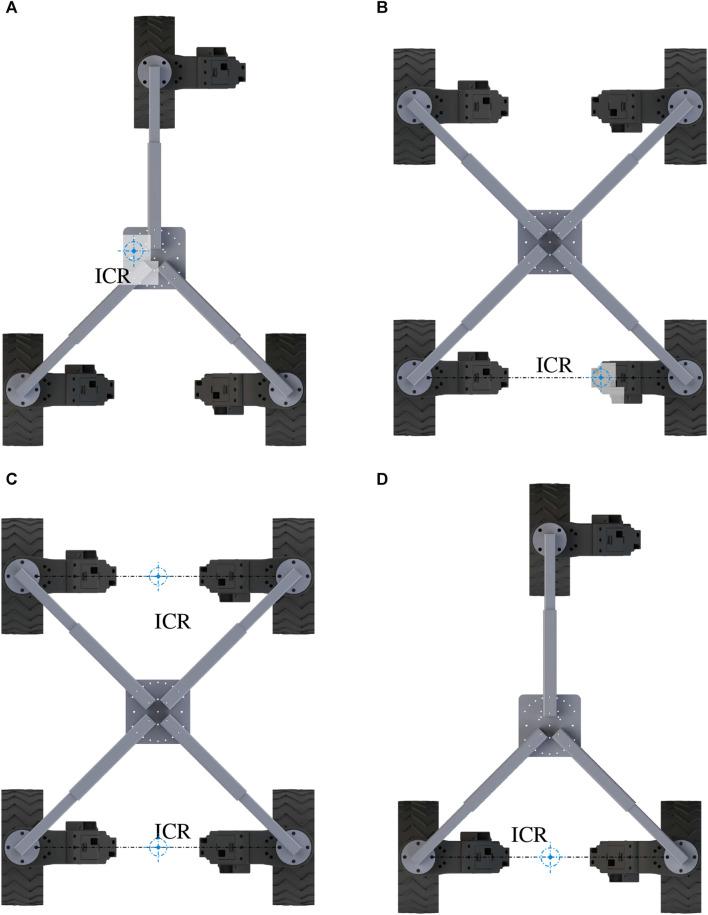
Clockwise from top left: **(A)** ICR placed centrally between all attached wheels, producing non-holonomic omnidirectional configuration. **(B)** ICR placed in line with the drive axis of the rear two wheels of a four-wheeled platform, confining the platform to Ackerman control. **(D)** ICR placed in line with the drive axis of the back two wheels of a three-wheeled platform, restricting the platform to tricycle control. **(C)** ICR located between both sets of wheels produces differential drive or skid steer. Image reproduced from Cordie et al. (2019a).

The controller designed for the NeRobot modular robot system is central to its ability to reconfigure quickly and redeploy. It is based on a parametric robot model that, once modified, propagates through the remainder of the system and generates the controller. The generated controller maintains body velocity by calculating the relative velocity of each wheel. Each wheel independently maintains its own desired velocities and heading, to illustrate Figure 3 shows three NeWheels achieving the desired linear and angular body velocity. The implementation of a velocity controller allows LR through the movement of the nominal center of the platform or ICR. Moving the ICR produces a different platform behavior from the same configuration with the same input velocities, emulating multiple motion models. Locating the ICR centrally between the wheels attached to the platforms allows non-holonomic omnidirectional motion (Figure 4A). The ICR located on the axis of rotation of the rear wheels, by restricting the control input to [*x*,*θ*]^*T*^, produces a platform with Ackerman steering (Figure 4B). Similarly, a tricycle model is produced in platforms with three wheels, and the ICR is placed between the rear pair (Figure 4D). Finally, by configuring ICR between both pairs of wheels, the platform becomes a differential drive or skid steer (see the bottom left of Figure 4C).

#### 3.2.1 Controller adaptation

The forward kinematics detailed in Equation 5 enables LR through the shifting of the ICR, creating the control models (Figure 4). We now differentiate between the failed and functional actuators, enabling the forward kinematics to deal with actuator failures. This revision is seen in changes to the *J*_1_ and *J*_2_ matrices (Eqs 6 and 7). Under regular operation, the steered wheels *J*_1_(*β*) of each of the NeWheel module comprise the time-varying function *J*_1_ (*β*_*s*_), updating the rotation of the wheels at each time step (Eq. 1). Wheels with a failed steering actuator have the corresponding *J*_1_(*β*) row replaced, with *J*_1_ (*β*_*f*_) representing a fixed joint (Eq. 2). The variable *ϕ* describes the state of the drive motor for each NeWheel module (Eq. 7). A functional time-varying wheel actuator is noted as $\dot{\phi }$, and ***ϕ***_***f***_ indicates a failed actuator. The NeRobot *NR* state is now a set of NeWheel modules $NW\;NW_{n}={\left[\begin{array}{cccc} \beta & \phi & x & y \end{array}\right]}^{T}$ . The example (NR 3) shows that *NW*_1_ is functional, while *NW*_2_ shows a failed steering actuator, *NW*_3_ shows a failed drive actuator, and *NW*_*n*_ represents a failure of both the steering and drive actuators, known as a failed module.

$$\begin{array}{lll} \begin{array}{ccc} & J_{1}\left(\beta_{s}\right)= & \left[\begin{array}{ccc} \sin\left(\theta_{b1}+\beta_{sn}\right) & -\cos\left(\theta_{b1}+\beta_{sn}\right) & -l\cos\left(\beta_{sn}\right) \end{array}\right], \end{array} & & \end{array}$$ (1)

$$\begin{array}{lll} \begin{array}{ccc} & J_{1}\left(\beta_{f}\right)= & \left[\begin{array}{ccc} \sin\left(\theta_{b1}+\beta_{fn}\right) & -\cos\left(\theta_{b1}+\beta_{fn}\right) & -l\cos\left(\beta_{fn}\right) \end{array}\right], \end{array} & & \end{array}$$ (2)

$$NR=\left[\begin{array}{ccccc} \beta_{1s} & \beta_{2f} & \beta_{3s} & \ldots & \beta_{nf}\\ \dot{\phi_{1}} & \dot{\phi_{2}} & \phi_{3f} & \ldots & \phi_{nf}\\ x_{1} & x_{2} & x_{3} & \ldots & x_{n}\\ y_{1} & y_{2} & y_{3} & \ldots & y_{n} \end{array}\right].$$

The software application developed for the NeWheel system allows the physical and simulated systems to be reconfigured both before and after deployment. Central to this ability is the parametric model developed for the NeWheel system. At the run time, this model is loaded into the parameter server. Parameters describing the location of individual wheels and the sensor packages are loaded from the file or input through a simple GUI. This robot model propagates throughout the remainder of the software application used for visualization, collision checking, and updating the kinematic model and sensor locations. The remainder of the software system is divided into three sections: a core or central controller, individual wheels, and the planner. The core provides the link between the planner and the individual wheels. It accepts inputs as desired body velocity while outputting the desired velocity for individual modules. With the relative location of individual modules loaded into the parameter server, the principles of rigid body motion are used to transform the desired body velocity such that

$$v_{w}i=v_{B}+\omega_{B}\times r_{wi/B},$$ (3)

$$\alpha_{w}i=\alpha_{B}+\omega_{B}^{2}\times r_{wi/B}+\omega_{B}\times \left(\omega_{B}\times r_{wi/B}\right).$$ (4)

Here, *v*_*B*_ is the velocity of the body, *ω*_*B*_ is the angular velocity of the body, and *r*_*wi*/*B*_ is the distance from the center of rotation to the center of individual wheels (Eq. 3). *α*_*B*_ is the acceleration of the body, and *v*_*w*_ *i* and *α*_*w*_ *i* are the velocity and acceleration of individual wheels, respectively (Eq. 3). Wheel odometry can be used to estimate the pose of the platform with no additional sensors. *ζ*_*O*_ is the location of the platform in the odometry frame and is calculated by integrating the body velocity with respect to time (Eq. 5).

$$\zeta_{O}=\int R{\left(\theta \right)}^{-1}J_{1}{\left(\beta_{s}\right)}^{-1}J_{2}\dot{\phi }dt.$$ (5)

*R*(*θ*)^−1^ is the homogeneous transform matrix between the robot’s pose and the odometry frame. *J*_1_ (*β*_*s*_) is the *n* × 3 matrix, with each row containing the kinematic constraints of a steered wheel with a pseudo-inverse taken to achieve $J_{1}{\left(\beta_{s}\right)}^{-1}$ . Here, *θ*_*bn*_ is the angle of the body to the wheel’s frame of reference, and *θ*_*wn*_ is the heading of individual wheels (Eq. 6).

$$J_{1}\left(\beta_{s}\right)=\left[\begin{array}{ccc} \sin\left(\theta_{b1}+\theta_{w1}\right) & -\cos\left(\theta_{b1}+\theta_{w1}\right) & -l\cos\left(\theta_{w1}\right)\\ \sin\left(\theta_{b2}+\theta_{w2}\right) & -\cos\left(\theta_{b2}+\theta_{w2}\right) & -l\cos\left(\theta_{w2}\right)\\ \vdots & \vdots & \vdots \\ \sin\left(\theta_{bn}+\theta_{wn}\right) & -\cos\left(\theta_{bn}+\theta_{wn}\right) & -l\cos\left(\theta_{wn}\right) \end{array}\right],$$ (6)

$$J_{2}\dot{\phi }=\left[\begin{array}{cccc} r_{1} & 0 & \ldots & 0\\ 0 & r_{2} & \ldots & 0\\ \vdots & \vdots & \ddots & \vdots \\ 0 & 0 & \ldots & r_{n} \end{array}\right]\left[\begin{array}{c} \dot{\phi_{1}}\\ \dot{\phi_{2}}\\ \vdots \\ \dot{\phi_{n}} \end{array}\right].$$ (7)

Here, *J*_2_ is an *n* × *n* diagonal matrix of the wheel radius *r*_*n*_, and $\dot{\phi }$ is a column vector *n* in the length of wheel angular velocity $\dot{\phi_{n}}$ (Eq. 7). This approach to odometry assumes an ideal surface contact and no slip in the system. Improvements on these results have been achieved using standard laser odometry packages that fuse wheel odometry data.

Individual NeWheel modules work as follows: the desired velocity commands are received from the core, and the modules' internal control loop maintains the desired heading and velocity while returning telemetry to the core. This telemetry includes the motor position and velocity used for calculating odometry, drawing current, and diagnostic feedback. The base planner is an abstracted input, with the only requirement being a body velocity output.

### 3.3 Design reconfiguration strategy

**FIGURE 5:**
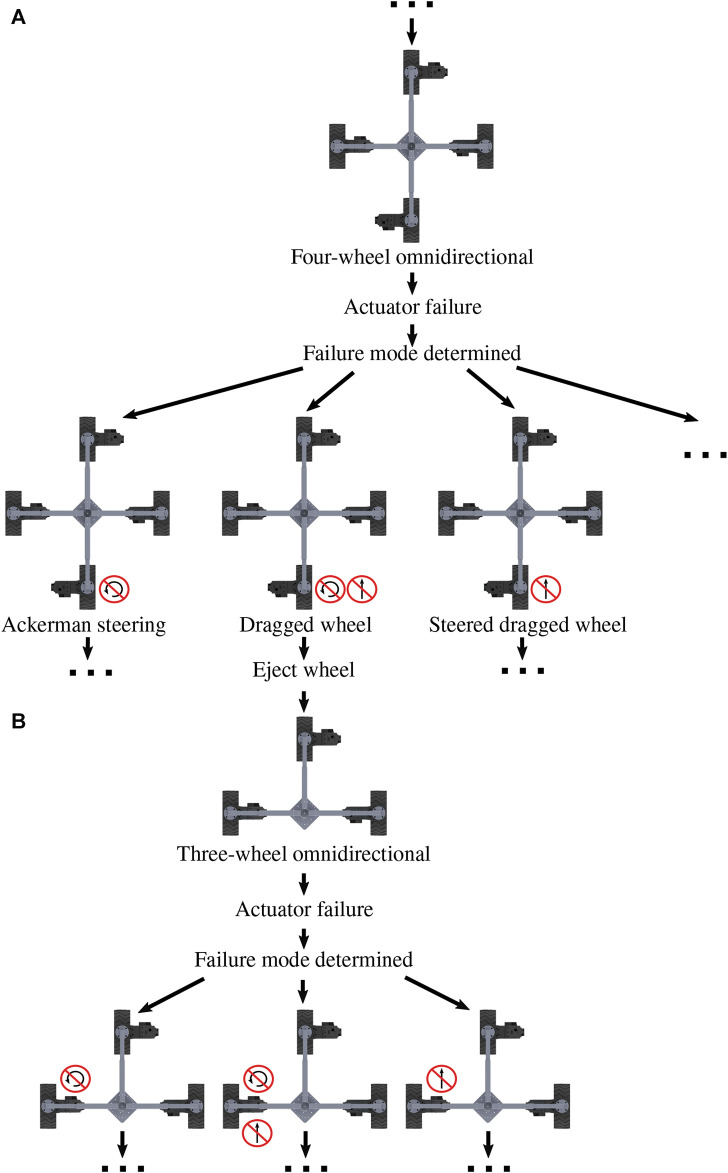
**(A)** Partial diagram of a four-wheeled modular robot adapting to the point of ejecting a wheel. **(B)** Followed by the failure of a module in the three-wheeled platform. Image reproduced from Cordie et al. (2019a).

Figure 5 is a decision tree for the transition from a robot with four fully-functional NeWheel modules ( $C_{a_{4}}$ ) to a partially functional three-wheeled ( $C_{a_{3}}$ ) robot. Here, $C_{j_{k}}$ describes the NeRobot configurations, j denotes the robot body type, and k indicates the number of NeWheels attached. Failure responses have been explored for the four- and three-wheeled systems as they have the least ability to reconfigure by removing a wheel. These two configurations, $C_{a_{4}}$ and $C_{a_{3}}$, reflect the last remaining modules able to operate as a robot isolated from repair fails. The proposed strategy retains partially damaged or broken modules by implementing LR and adapting the control strategy based on the hardware available. Reading from the top, Figure 5 proposes an omnidirectional control strategy for a platform with all modules functioning. The appropriate motion model is adopted as failure modes are detected. The dragged-wheel strategy is implemented in the case of failed steering and drive actuator. If a NeWheel becomes a liability, the robot can become an n-1 system by ejecting the affected module. After module ejection, the robot becomes a three-wheeled platform, $C_{a_{3}}$ , capable of omnidirectional motion. The resulting three-wheeled platform is capable of accommodating future failures before the platform fails. Edge cases for this proposed strategy are numerous and not explored in this work as a situation, and platform-dependent solutions would be required. We have explored three approaches to failure adaptation.

• Fixed configuration (FC): it is indicative of a robot with a fixed morphology and fixed control configuration and cannot accommodate a failure. This is considered a baseline typical of many robots.
• Locomotion reconfigurability (LR): it is indicative of a robot with a fixed morphology and reconfigurable controller, and the robot switches locomotion styles to accommodate actuator failure.
• Design reconfigurability (DR): it is indicative of a robot with reconfigurable morphology and controller, and the robot can eject a failed module and switch locomotion styles to accommodate actuator failure.

This work has explored locomotion reconfigurability (LR) and design reconfigurability (DR) as alternatives to robots deployed with a traditional fixed configuration (FC). The controller’s internal model is updated during configuration changes based on the robot’s last known functional state. The controller is updated with an idealized kinematic model based on this state, as described in Section 3.2. We do not perform any parameter tuning for the default individual wheel controllers as the robot morphology changes. We found that the default wheel controllers (PID) tracking the desired velocity are robust enough to maintain the whole-body velocity in the field.

We have tested DR on both the NeRobot and the simulated NeRobot. To affect the DR of the physical NeRobot, we removed a pin and let the affected actuator fall away. Meanwhile, the simulated NeRobot had the affected actuator removed from the model. Future iterations of the NeRobot would explore other opportunities for positive mechanical separation, of which there are many. Examples of positive mechanical separation that we could explore further include a spring and pin puller, magnetic connections, and explosive bolts.

### 3.4 Locomotion reconfigurability (shifting the ICR)

A NeRobot platform is capable of non-holonomic omnidirectional motion when all connected modules are functional. The robot states NR 4 and NR 5 show examples of configurations with three and four NeWheel modules, respectively. In configurations with all the modules functioning, the ICR is located centrally between the NeWheel modules. This freedom of movement allows maneuvers such as orientating itself to the goal and heading as it approaches the target.

$$NR=\left[\begin{array}{cccc} \beta_{1s} & \beta_{2s} & \beta_{3s} & \beta_{4s}\\ \dot{\phi_{1}} & \dot{\phi_{2}} & \dot{\phi_{3}} & \dot{\phi_{4}}\\ x_{1} & x_{2} & x_{3} & x_{4}\\ y_{1} & y_{2} & y_{3} & y_{4} \end{array}\right]$$

or

$$NR=\left[\begin{array}{ccc} \beta_{1s} & \beta_{2s} & \beta_{3s}\\ \dot{\phi_{1}} & \dot{\phi_{2}} & \dot{\phi_{3}}\\ x_{1} & x_{2} & x_{3}\\ y_{1} & y_{2} & y_{3} \end{array}\right].$$

**FIGURE 6:**
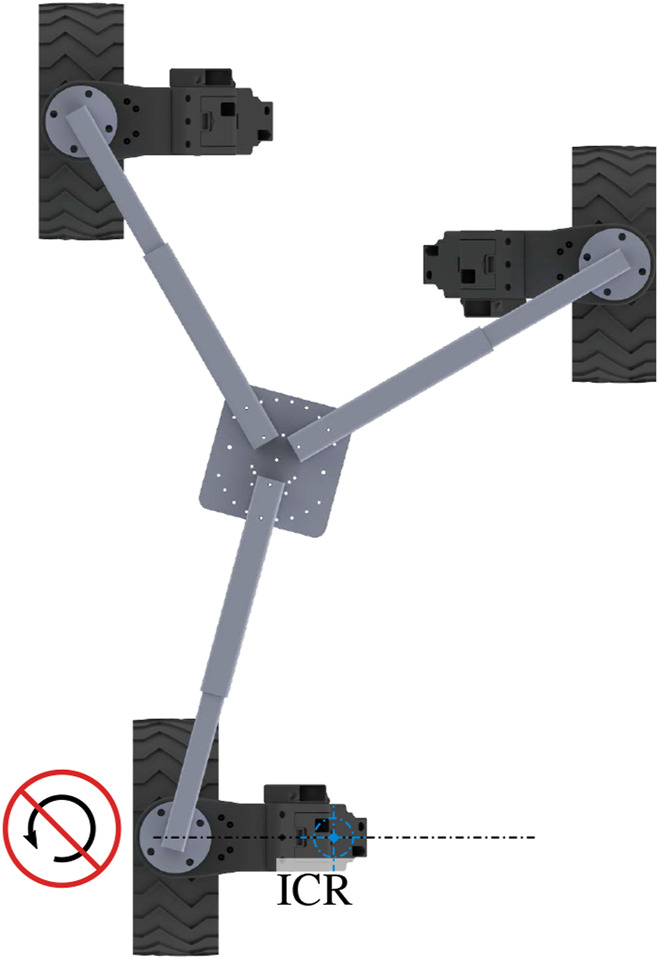
Robot configured to align with the failed steering actuator on the back of a three-wheeled platform.

The failure of a steering actuator, as seen in robot configurations NR 6 and NR 7 on a robot platform deployed with NeWheels, removes a degree of freedom from the platform. This failure prevents the platform from moving perpendicular to the heading of the damaged wheel. Locomotion reconfigurability implements an Akerman style motion model to accommodate the loss functionality, minimizing the impact on the robot. To achieve this change in the controller, the robot’s ICR is moved to a point along the failed module’s drive wheel axis of rotation. When the center is relocated, the symbolic front of the platform passes through the new center point parallel to the heading of the affected wheel (Figure 6). The remaining NeWheels maintain their full functionality by adopting the required heading for driving and steering. Restricting control input for the *y*-axis (lateral motion) and remapping it as angular velocity result in the platform rotating on the spot to orientate the platform in the desired direction. This functionality is typically not seen in car-like robots.

$$NR=\left[\begin{array}{cccc} \beta_{1s} & \beta_{2s} & \beta_{3f} & \beta_{4s}\\ \dot{\phi_{1}} & \dot{\phi_{2}} & \dot{\phi_{3}} & \dot{\phi_{4}}\\ x_{1} & x_{2} & x_{3} & x_{4}\\ y_{1} & y_{2} & y_{3} & y_{4} \end{array}\right]$$

or

$$NR=\left[\begin{array}{ccc} \beta_{1s} & \beta_{2s} & \beta_{3f}\\ \dot{\phi_{1}} & \dot{\phi_{2}} & \dot{\phi_{3}}\\ x_{1} & x_{2} & x_{3}\\ y_{1} & y_{2} & y_{3} \end{array}\right].$$

**FIGURE 7:**
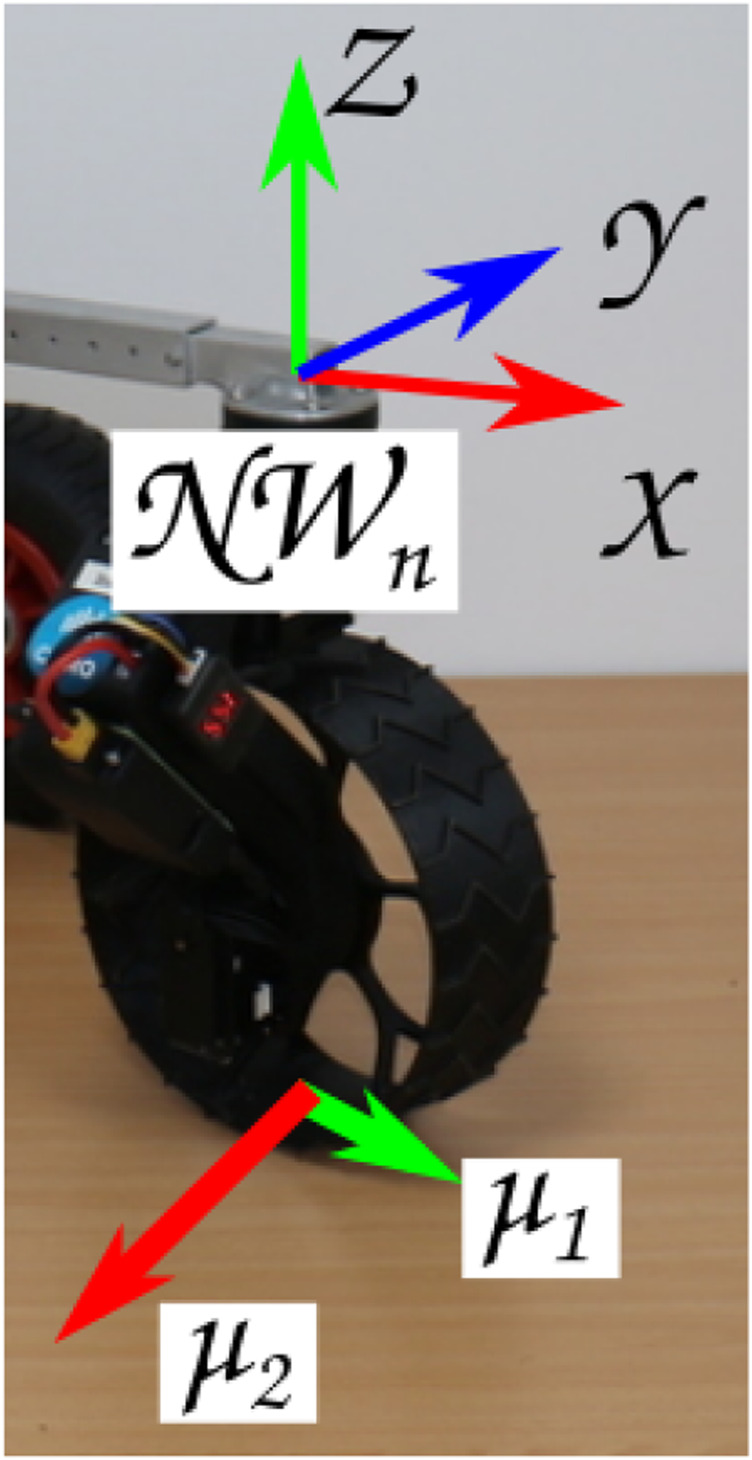
Assumed friction when dragging an incapacitated NeWheel.

A steerable module with a failed drive actuator could take many forms, but the following assumptions are made for the purpose of these experiments. The drive motor is locked in place with no or minimal ability to move, while the steering motor has retained full functionality. The friction model of the wheel/surface (Figure 7) is such that dragging the wheel perpendicular to the axis of rotation incurs the least friction penalty. Similar to the failed steering actuator mentioned above, the location of the failed drive actuator in the robot states NR 8 and NR 9 is only indicative due to the system’s ability to re-orientate.

$$NR=\left[\begin{array}{cccc} \beta_{1s} & \beta_{2s} & \beta_{3s} & \beta_{4s}\\ \dot{\phi_{1}} & \dot{\phi_{2}} & \dot{\phi_{3}} & \phi_{4f}\\ x_{1} & x_{2} & x_{3} & x_{4}\\ y_{1} & y_{2} & y_{3} & y_{4} \end{array}\right]$$

or

$$NR=\left[\begin{array}{ccc} \beta_{1s} & \beta_{2s} & \beta_{3s}\\ \dot{\phi_{1}} & \dot{\phi_{2}} & \phi_{3f}\\ x_{1} & x_{2} & x_{3}\\ y_{1} & y_{2} & y_{3} \end{array}\right].$$

**FIGURE 8:**
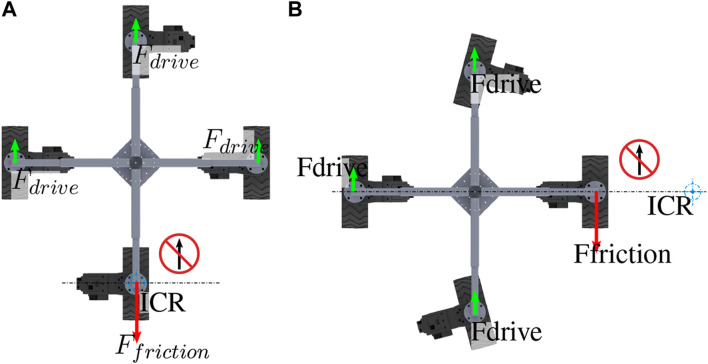
Four-wheeled configuration as per the NeRobot model NR 8 with a failed drive actuator; **(A)** shows the platform re-orientated to travel without angular velocity, and **(B)** depicts the platform rotating.

The platform retains the ability to re-orientate the failed module under the conditions of a single drive actuator failure. If the desired velocity has only a linear component and no angular velocity, LR orientates the platform and updates the model NR 8, locating the failed module among the remaining modules, as shown in Figure 8. Therefore, the resulting force from the dragged wheel is distributed between the remaining modules. Equation 8 gives the force required to overcome the force induced on the system by the failed wheel, where *F*_*f*_ is the force created by the failed wheel dragging and $F_{d_{i}}$ is the individual force of the functional modules.

**FIGURE 9:**
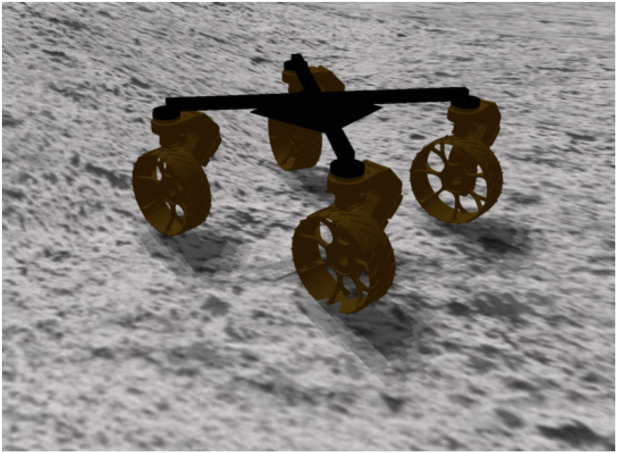
NeWheel platform depicted in a Gazebo simulation environment.

When rotating, the platform’s ability to shift the ICR allows a platform with a failed drive actuator to shift the ICR outside the body of the robot. Shifting the ICR away from the failed wheel by updating the model in NR 8 allows management of the torque required of the functional wheels (Figure 8). This system behavior is captured by Equation 9, where the torque on the system generated by the dead wheel *F*_*f*_ × *r*_*f*_ is counteracted by the torque created by the remaining wheels $F_{d_{i}}\times r_{d_{i}}$ . Here, *F*_*f*_ is the force generated by dragging the dead wheel, *r*_*f*_ is the radius from the ICR to the failed wheel, $F_{d_{i}}$ is the force generated by individual functional wheels, and $r_{d_{i}}$ is the radius from the ICR to the individual functional wheels.

$$F_{f}=\overset{n}{\underset{i=1}{\sum }}F_{d_{i}}$$ (8)

$$F_{f}\times r_{f}=\overset{n}{\underset{i=1}{\sum }}F_{d_{i}}\times r_{d_{i}}$$ (9)

The two scenarios above assume that the remaining wheels can overcome the forces generated by the failed wheel dragging. The remaining wheels must still produce a functional platform for this strategy to be viable. In the scenario where dragging the failed wheel jeopardizes the deployment, the option to perform DR and eject the module must be considered.

The failure of a drive and steer actuator in the same module leaves the platform with a fully incapacitated module (see robot models NR 10 and NR 11). Unlike the steered dragged wheel, the platform must then reconfigure using the remaining wheels to minimize the impacts of dragging the nonfunctional wheel. This scenario leaves the platform unable to distribute the forces among the remaining wheels evenly. LR is still attempted before implementing DR.

$$NR=\left[\begin{array}{cccc} \beta_{1s} & \beta_{2s} & \beta_{3s} & \beta_{4f}\\ \dot{\phi_{1}} & \dot{\phi_{2}} & \dot{\phi_{3}} & \phi_{4f}\\ x_{1} & x_{2} & x_{3} & x_{4}\\ y_{1} & y_{2} & y_{3} & y_{4} \end{array}\right]$$

or

$$NR=\left[\begin{array}{ccc} \beta_{1s} & \beta_{2s} & \beta_{3f}\\ \dot{\phi_{1}} & \dot{\phi_{2}} & \phi_{3f}\\ x_{1} & x_{2} & x_{3}\\ y_{1} & y_{2} & y_{3} \end{array}\right].$$

## 4 Experimental results

We performed two categories of experiments: simulation of full platform failure and the effect of performance for a given actuator failure, both on simulation and real hardware.

Algorithm 1 Robot goal-seeking with failure evaluation.

 **Input**: goalPosition, locomotionStrategy
 **Output**: Success or Failure state
 1 **Procedure** GoToGoal ():
  2 **While** *not AtGoalPosition* do
    3 Use to move toward goalposition **if** EvaluateFailurestate () **then**
     4 **return** Failure State
    5 **return** Success State
 **6 Function** EvaluateFailureState ():
   7 sample ← RandomVariable () if sample ≤ ProbabilityofFailureRate **then**
   8 **return** True
  9 **else**
   10 **return** False

### 4.1 Simulation of full failure

The NeRobot simulated in Gazebo consisted of four NeWheels connected via simulated links in a symmetric 0.6 × 0.6 m configuration similar to the robot seen in Figure 9 the robot was configured for omnidirectional motion. The simulated robot was given random waypoints sequentially while sampling a failure event from a probability of 0.001 per second in simulation time. The simulator ran a total of 550 times, randomly producing failures until the robot was immobile. Each actuator within the NeRobot had a failure probability of 0.001 every second of simulation. The failure rate of *ρ* = 0.001 represents a compromise balancing the time taken to simulate failure with the distance travelled. Simple failure models responded linearly to changes in failure rate, and this assumption is used for the Gazebo simulation. Before beginning each configuration/approach instance, the model is checked for kinematic viability in three areas. First, the center of mass is within the support polygon; second, the failed steering actuators do not reduce the platforms degrees of freedom; and finally, confirming that more than 50% of the drive actuators is functional. After confirming robot viability, the platform is given a list of goals to drive toward. As the robot drives between the goals, each actuator is sampled with the constant failure rate. The failure of an actuator triggers the controller reconfiguration and simulation branch. Each simulation continues until no further viable configurations can be found. Data regarding the robots pose, goals passed, joint velocity, effort, and position are collected. A pseudocode is presented in Algorithm 1 for running the simulated failure rate experiment for the three locomotion strategies FC (fixed control), LR (locomotion reconfiguration by changing the center of rotation), and DR (design reconfiguration by possibly ejecting the failed module using the decision tree 5).

### 4.2 Performance evaluation for simulated actuator failures

The NMRS was used for robot testing of the three approaches proposed in this work. These tests were carried out using three different robot configurations on a NeRobot with four NeWheels in a symmetric 0.6 × 0.6-m configuration, namely, a platform with all actuators functional as a baseline, a second with a failed steering actuator, and a third with a failed drive actuator. The fully functional platform was placed at the test origin and driven to a goal at [5.0 m, 5.0 m, 3.14*rad*]^*T*^ to provide a baseline. The platform was then reconfigured to simulate a failed steering actuator. FC tested no controller or morphology change. LR saw the center of rotation shifted to the axis of the failed module. DR tested ejecting the failed module and operating as a three-wheeled platform. The tests saw the platform drive to the goal at [5.0 m,5.0 m,3.14*rad*]^*T*^. This process of framework testing was repeated with the failed drive actuator. Testing in this manner was repeated in two environments: a warehouse floor and in leaf litter on a hill (see Figure 1).

### 4.3 Experimental results

The ability to quickly change the modules within a modular robot allows an operator to repair a deployed robot in the field. This work argues that the same modular robotic strategies that enable an operator to assemble and use a robot quickly facilitate the improved robustness of the same robots. The results show that moving the robot’s ICR creates multiple motion models from a single robot configuration. This then allows the controller to handle failed actuators, enabling the robot to continue. At the same time, modular design practices allow immobilized robots to eject failed or trapped components and continue operation.

### 4.4 Qualitative result of locomotion reconfigurability

**FIGURE 10:**
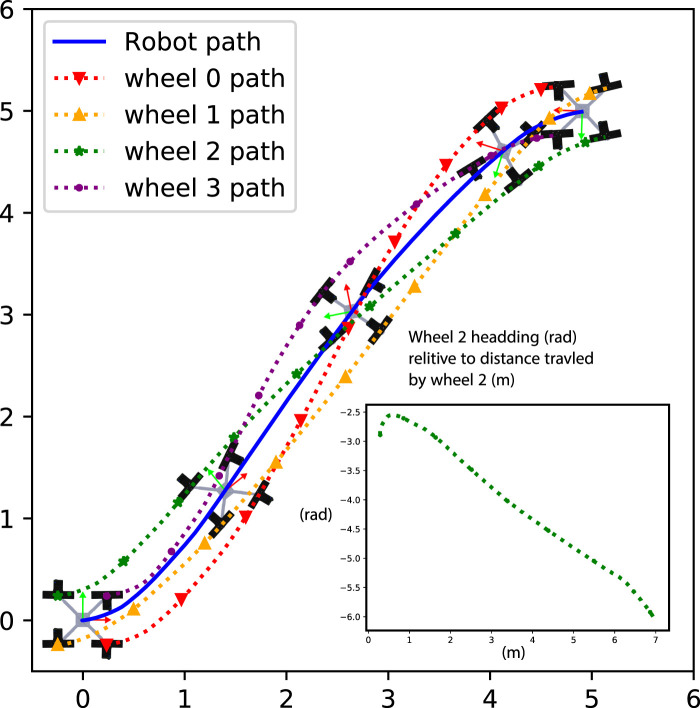
Path travelled by a functional NeRobot.

**FIGURE 11:**
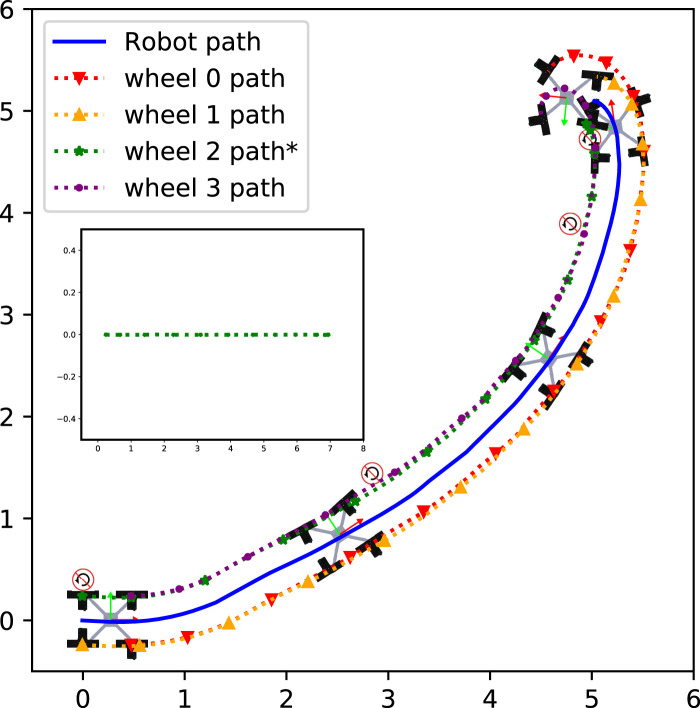
Path travelled by an NeRobot with a failed steering actuator.

Deployment of the $C_{a_{4}}$ NeRobot with failed actuators in simulation gave each robot the goal position of [5.0 m, 5.0 m, 3.14 *rad*]. The NeRobot with four fully functional NeWheel modules drove straight to the goal, rotating its orientation as it travelled (Figure 10). The experiment was repeated with the same $C_{a_{4}}$ NeRobot after suffering a steering actuator failure (Figure 11). The robot travels to the goal, pose looping around to the goal. This experiment showed that the robot was capable of surviving failed actuators and driving to a goal.

### 4.5 Simulated robot experiments

The 550 Gazebo simulations tested the robots for failure using the framework described in Section 3. After an actuator failure, FC continues with no change, LR adapts the center of rotation to the failure, as shown in Figure 5, and DR ejects the failed module and reconfigures the controller.

#### 4.5.1 Impact on the average distance traveled

**FIGURE 12:**
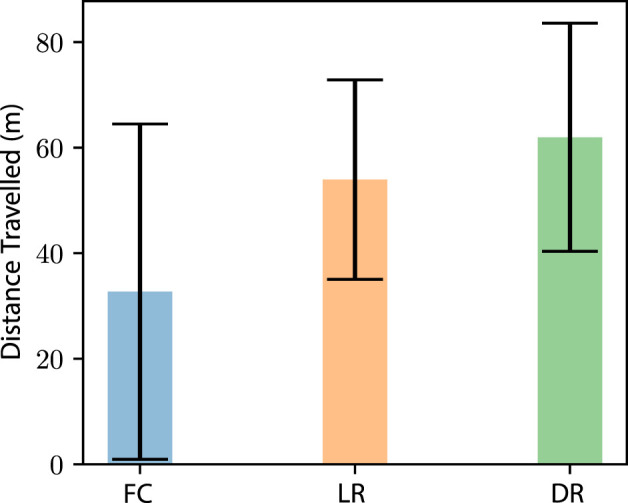
Average distance travelled and one standard deviation of each approach implemented (*n* = 550). Distances are Euclidean distances to goals passed. The three approaches are fixed configuration (FC), locomotion reconfigurability (LR), and design reconfigurability (DR).

The first experiment explored the average distance traveled by a NeRobot implementing each of the proposed policies (Figure 12). In this scenario, LR outperformed FC by 64%. The DR experiment saw an improvement of 89% when compared with FC and 14% improvement when compared with LR. In rejecting the null hypothesis that not adapting to actuator failure results in a greater average distance travelled, a *p*-test comparing each data set was calculated. With the results of each experiment returning *ρ* < 0.001, there is a significant reason to reject the null hypothesis and accept that adapting to actuator failure improves the distance travelled by the robot.

**FIGURE 13:**
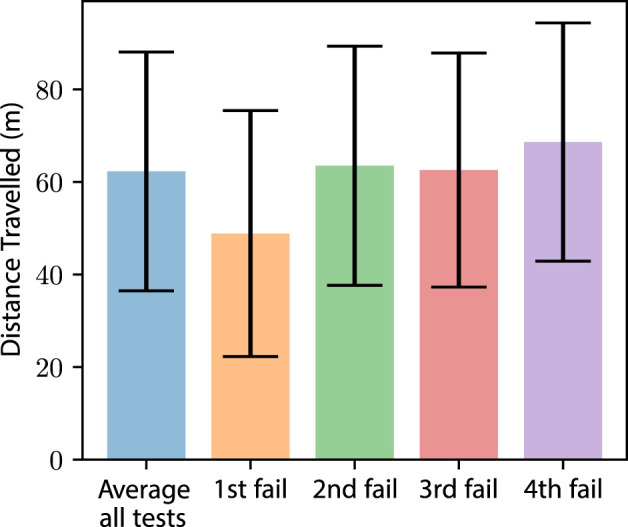
Average distance travelled and one standard deviation (*n* = 550) of the robot before platform failure. Each dataset shows the number of actuator failures before module ejection.

With the benefits of allowing the robot to eject a failed module on platform longevity (Figure 12), the timing of this ejection versus adoption is explored. This experiment tested the robots for failure, with each instance branching after a failure. After branching, we tested each response to each failure, FC, LR, and DR. This approach allowed us to test each permutation of ejection timing. The analysis of these data showed us that ejecting a module after the robot suffers a single failure is detrimental to the robot’s longevity (Figure 13), decreasing the distance travelled by 21%. Similarly, if a robot can continue to operate until suffering a fourth failure, the distance travelled is increased by 10%. These data indicate that a deployed robot should not eject a module until not ejecting the module would jeopardize the deployment.

The data show that a robot, or rovers like Spirit and Opportunity, with the ability to adapt to failure, increases robustness to actuator failure. These results were found to be true when comparing a fixed-configuration robot to a robot that can reconfigure its locomotion. Further improvements in robustness were seen in robots that are able to eject modules with failures.

#### 4.5.2 Impact on motion effort

**FIGURE 14:**
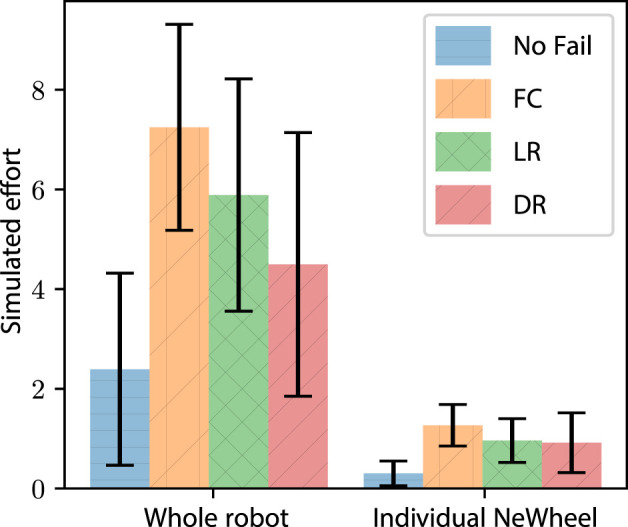
Simulation experiment: the average instantaneous effort and one standard deviation (*n* = 550). The average instantaneous effort and one standard deviation (*n* = 550) while implementing FC, LR, and DR. The data are separated into the whole robot and individual modules.

Another effect of actuator failure on the rover Spirit was the increased effort when operating on Mars with a failing actuator (Townsend et al., 2014). The effects of the FC, LR, and DR approaches on the robot and module effort (simulated as torque values for the actuators) were explored. We recorded the average effort used by each of the policies, with data separated into a whole robot and average module (Figure 14). Each of the evaluated policies reported increased effort when compared with a baseline robot without failure, echoing the reports of increased effort from Spirit after failure. The LR and DR policies did show a decrease in effort when compared with FC by 18% and 38%, respectively. Although DR showed a 24% decrease in effort when compared to LR. Further inspection of the LR/DR comparison shows similar effort at a module level, suggesting that the effort decrease is due to the ejection of the module. This exploration of simulated effort shows that both LR and DR policies reduced the effort in the simulated robot when compared to the FC approach.

The use of LR and DR increased the distance travelled and reduced the effort of the simulated robots. From the results, two conclusions can be drawn. First, if the overall distance traveled by the robot is the most important aspect, then retaining modules as long as possible provides the best results. Second, if on-board power is limited, ejecting a failed module is more energy-efficient than retaining failed modules. The use of LR and DR is shown to benefit a deployed robot with the scenario dictating the appropriate implementation.

### 4.6 Physical robot evaluation

**FIGURE 15:**
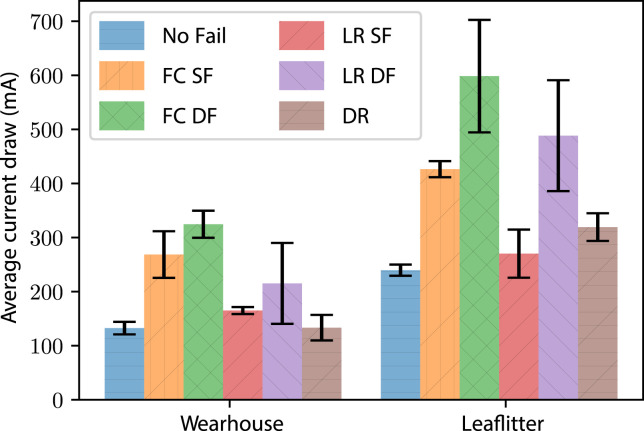
Real-world experiment: average current drawing and one standard deviation (*n* = 12) of a robot during the evaluation of the different approaches. Average current drawing and one standard deviation (*n* = 12) of a robot during the evaluation of the different approaches of FC, LR, and DR. Demonstrations of two failure types were conducted: steering failure (SF) and drive failure (DF) in both the warehouse and the leaf litter environments.

**FIGURE 16:**
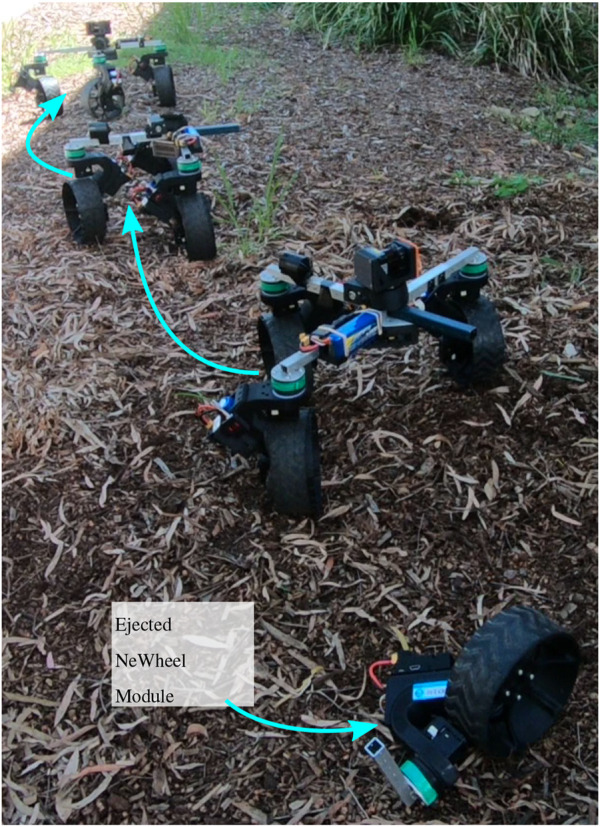
NeRobot demonstrating the concept of module ejection when employing design reconfigurability. Note that in this sequence of overlapping images, we see the robot realigning the center of rotation (ICR) to better suit the direction of motion. The active decoupling of the module was done manually but can be implemented by any mechanical latch, magnetic latch, or any other suitable mechanisms depending on the robot design.

To validate the three approaches implemented in the Gazebo simulation, we evaluated the policies on a physical twin assembled using the NMRS. The experiments were carried out in two different environments: a warehouse with a level floor and on the side of a hill in leaf litter. The three robot configurations used in each environment were no actuator failure, steering failure (SF), and drive failure (DF). The experiments showed that each of the approaches, FC, LR, and DR, created functional robots and validated the results of the simulated effort experiments. Evaluation of the approaches showed that similar to the simulations, FC drew the most current when compared with the other approaches for all configurations in both environments (Figure 15). Furthermore, the FC approach became bogged when demonstrating both SF and DF in the leaf litter, resulting in an incapacitated robot. When operating in the warehouse, the LR approach operated with 25% less efficiency in the SF configuration than the functional robot and 62% less efficiency in the DF configuration. When operating in the leaf litter, the robot implementing LR with a SF reached the goal using 13% more current than the fully functional robot. However, the robot with the DF failed to move in the leaf litter using the LR approach. This failure provided an opportunity to implement DR on a robot incapable of continuing by ejecting a module (Figure 16). Unlike the warehouse experiments, DR operating in the leaf litter drew more current than the ground truth. The increased drawing of the current is attributed to the additional proportional load on each module and the incline of the leaf litter course. Each of the three approaches, FC, LR, and DR, has been shown to function on a physical modular robot. Once again, LR and DR drew less current than FC, and it demonstrated a robot ejecting a failed module and continuing to operate.

Simulated experiments with the NeRobots have shown that robots capable of adapting to failure outperform their fixed configuration counterparts. Furthermore, robots that could eject modules and reconfigure their design travelled further than robots that could only reconfigure their controller. However, the results also showed that it is preferential to retain modules while their failures can be adapted to as early ejection reduces the distance travelled by the robot. As well as the improved distance travelled, it was shown that adaptation to actuator failure decreased the effort used by the robot when operating with failures. The results of these simulated experiments show that the preferred approach for platform longevity depends on the desired outcome of the deployment. If distance travelled is the most important factor, modules should be retained as long as possible using LR. If the platform has tight power restrictions, then ejecting the failed module using DR would be the best approach. Experiments with the physical robot validated the proposed approaches and showed a decrease in the current drawn by the robot when using LR to adapt to actuator failure. When operating in the leaf litter, the NeRobot was able to eject a module with a failed drive actuator and continue operating after becoming bogged.

## 5 Conclusion

Field robots built with a single configuration or morphology have the same limiting controllers when deployed. The operator has limited options to continue the mission in the case of actuator failure or if the robot becomes immobile. In the instance of a failed actuator, the robot can attempt to overcome the failure with the remaining working actuators. This brute force approach requires the remaining actuators to work harder, thus consuming more energy. Where possible, a revised controller could be provided to the robot. In the case of a robot trapped in its environment, the immobilized robot can only perform work where it sits.

The NeRobot modular robot system has the ability to quickly change controllers. This functionality allows the system to change the size, shape, and the number of modules in a deployed robot. This ability to change is key to the robot degrading gracefully. By using LR to shift the robot’s ICR, it can adapt to a failed actuator by changing the motion model. Adaptability to changes in the number of modules used by the system facilitates the robot ejecting failed modules.

A set of simulated and physical experiments were developed for demonstrating the integration of the proposed functionality. These experiments used the NeRobot modular robot system in its $C_{a_{4}}$ configuration. The three policies evaluated were as follows:

• Fixed configuration (FC): it is indicative of a robot with a fixed morphology and fixed control configuration and cannot accommodate a failure. This is considered a baseline typical of many robots.
• Locomotion reconfigurability (LR): it is indicative of a robot with a fixed morphology and reconfigurable controller, and the robot switches locomotion styles to accommodate actuator failure.
• Design reconfigurability (DR): it is indicative of a robot with a reconfigurable morphology and controller, and the robot can eject a failed module and switch locomotion styles to accommodate the actuator failure.

FC emulates a fixed configuration robot with a similarly fixed controller. LR allows a robot with a fixed body configuration to modify its controller’s configuration based on the status of its actuators. Finally, DR assumes a modular robot capable of ejecting modules and reconfiguring its controller. The simulated version of the experiment presented the robots with a list of goals to move between, and random sampling created simulated actuator failures. With each simulated failure, the simulation branched tested the effectiveness of each policy in the current situation. All three policies were demonstrated on a physical robot with simulated steering and then with drive actuator failure. The experiments were repeated in two different environments; a warehouse and on a hill covered in leaf litter.

Robots employing LR and DR travelled further than those employing FC when the policy choices of simulated robots were compared. The experiments also showed that robots capable of ejecting modules (DR) travelled further than robots that could not. The timing of module ejection also had an impact on robot longevity. Platforms that ejected modules early were penalized compared to those that retained the modules and adapted to actuator failure. In addition to increased longevity, robots employing LR and DR reduced the effort required at both the module and robot levels. Similar to the reduction in effort observed in the simulation, the physical robot had a reduction in the current drawn when using LR and DR to adapt to failure when compared to FC. The physical robot became bogged in leaf litter while demonstrating the $C_{a_{4}}$ robot with a failed drive actuator. This scenario resulted in a practical demonstration of DR as the robot ejected the failed module and continued to its goal, operating in the $C_{a_{3}}$ configuration.

### 5.1 Limitations and future work

Future iterations of the NeRobot modular robot system could explore the use of an automated configuration advisor, simplifying deployments. In its current iteration, an expert operator makes robot design decisions based on the deployment environment. Passing this design expertise on to a novice operator would require a significant amount of training. The configuration advisor would provide robot designs for implementation using information including the number of modules, terrain type, existing maps, and the expected duration of the deployment. The resulting automated design would allow a novice to assemble an NeRobot suitable for their unique deployment.

Failure to maintain kinematic stability due to ejecting an NeWheel module remained a deployment-ending event in our evaluation of modular field robot robustness. The deployment is not viable once the ejection of any further modules causes the center of mass to shift outside the robot’s support polygon. Our proposed solution to this failure mode is the inclusion of self-reconfigurable links on such deployments. Although sufficient modules remain to maintain stability, the inclusion of the self-reconfigurable links would allow a robot to reconfigure its support polygon. By allowing the platform to self-reconfigure, the deployment could be extended, avoiding failure due to platform instability.

With more capability, we would explore further failure modes. Additional modes of interest are failure to detect a failure or failure to eject a failed module correctly. With further exploration of failure modes, we would develop a probabilistic model of robot failures to guide responses to failure. This future model would also help separate the functionality of modularity vs. reconfigurability. Depending on the results of such research, we could reduce the complexity of the deployed system by either deploying a modular or reconfigurable robot in place of a modular, reconfigurable robot.

Our approach also assumes that the failures are sequential, and the path of the individual modules failing can be detected reliably. There are many scenarios where such assumptions are not valid. We are currently exploring machine learning approaches to learn the failure modes to trigger morphological changes.

This work shows that including modular robotic capabilities into field robotics has improved robustness by allowing a robot to reconfigure its controller to cope with a fault or eject a failed module. The results showed that retaining modules for as long as possible increased the distance the robot traveled, while reconfiguring the controller reduced the effort/power consumption of the robot. If distance traveled is crucial to a mission’s success, retaining modules is of key importance. However, a robot on a tight energy budget may opt for module ejection at the cost of longevity. This would be a robot and situation-dependent decision.

## Data Availability

The datasets presented in this article are not readily available because the data can be generated by an algorithmic implementation of the approach, as outlined in the article, and does not require a specific dataset for analysis. Requests to access the datasets should be directed to tirtha.bandy@csiro.au.

## References

[B1] Alamdari A. Krovi V. (2014). “Active reconfiguration for performance enhancement in articulated wheeled vehicles,” in 2014 ASME Dynamic Systems Controls Conference, San Antonio, TX, USA, October, 2014.

[B2] Alamdari A. Krovi V. N. (2016). Design of articulated leg–wheel subsystem by kinetostatic optimization. Mech. Mach. Theory 100, 222–234. 10.1016/J.MECHMACHTHEORY.2016.02.010

[B3] Alamdari A. Zhou X. Krovi V. N. (2013). “Kinematic modeling, analysis and control of highly reconfigurable articulated wheeled vehicles,” in 37th Mechanisms and Robotics Conference (ASME), Portland, August, 2013. 10.1115/DETC2013-12401

[B4] Barrios L. Collins T. Kovac R. Shen W.-M. (2016). “Autonomous 6D-docking and manipulation with non-stationary-base using self-reconfigurable modular robots,” in 2016 IEEE/RSJ International Conference on Intelligent Robots and Systems (IROS), Daejeon, Korea (South), October, 2016, 2913–2919. 10.1109/IROS.2016.7759451

[B5] Bartlett P. W. Wettergreen D. Whittaker W. (2008). Design of the Scarab rover for mobility and drilling in the lunar cold traps. Int. Symposium Artif. Intell. Robotics Automation Space 3–6. 10.1184/R1/6552563.V1

[B6] Chen C.-A. Collins T. Shen W.-M. (2016). “A near-optimal dynamic power sharing scheme for self-reconfigurable modular robots,” in 2016 IEEE International Conference on Robotics and Automation (ICRA), Stockholm, Sweden, May, 2016, 5183–5188. 10.1109/ICRA.2016.7487724

[B7] Cordie T. Bandyopadhyay T. Roberts J. M. Steindl R. Dungavell R. Greenop K. (2016). “Enabling rapid field deployments using modular mobility units,” in Australasian Conference on Robotics and Automation (ACRA) 2016 (Australian Robotic and Automation Association), Canberra, Australia, December, 2015.

[B8] Cordie T. Steindl R. Dungavell R. Bandyopadhyay T. (2019a). “Modular field robots for extraterrestrial exploration,” in 70th International Astronautical Congress, Washington, D.C., USA, October 2019.

[B9] Cordie T. P. Bandyopadhyay T. Roberts J. Dunbabin M. Greenop K. Dungavell R. (2019b). Modular field robot deployment for inspection of dilapidated buildings. J. Field Robotics 36, 641–655. 10.1002/rob.21872

[B10] Das D. Banerjee S. Chernova S. (2021). “Explainable AI for robot failures: generating explanations that improve user assistance in fault recovery,” in Proceedings of the 2021 ACM/IEEE International Conference on Human-Robot Interaction, Boulder, CO, USA, March, 2021, 351–360. 10.1145/3434073.3444657

[B11] Davey J. Kwok N. Yim M. (2012). “Emulating self-reconfigurable robots - design of the SMORES system,” in 2012 IEEE/RSJ International Conference on Intelligent Robots and Systems, Vilamoura-Algarve, Portugal, October, 2012, 4464–4469. 10.1109/IROS.2012.6385845

[B12] Ha S. Kim J. Yamane K. (2018). “Automated deep reinforcement learning environment for hardware of a modular legged robot,” in 2018 15th International Conference on Ubiquitous Robots (UR), Honolulu, HI, USA, June, 2018, 348–354. 10.1109/URAI.2018.8442201

[B13] Hammond J. C. Biswas J. Guha A. (2019). Automatic failure recovery for end-user programs on service mobile robots. https://www.arxiv.org/abs/1909.02778.

[B14] Howe A. S. Wilcox B. Barmatz M. Voecks G. (2016). ATHLETE as a mobile ISRU and regolith construction platform. Pasadena, CA, USA: Jet Propulsion Laboratory, National Aeronautics and Space.

[B15] Jie Z. Shufeng T. Yanhe Z. (2009). Design and implementation of a modular self-reconfigurable robot. High. Technol. Lett. 15, 227–232.

[B16] Jing G. Tosun T. Yim M. Kress-Gazit H. (2016). An end-to-end system for accomplishing tasks with modular robots. Robotics Sci. Syst. XII. 10.15607/RSS.2016.XII.025

[B17] Kalouche S. Rollinson D. Choset H. (2015). “Modularity for maximum mobility and manipulation: control of a reconfigurable legged robot with series-elastic actuators,” in 2015 IEEE International Symposium on Safety, Security, and Rescue Robotics (SSRR), West Lafayette, IN, USA, October, 2015. 10.1109/SSRR.2015.7442943

[B18] Kassel S. (1971). Lunokhod-1 soviet lunar surface vehicle. Santa Monica, California, United States: RAND Corporation.

[B19] Kelly A. Seegmiller N. (2015). Recursive kinematic propagation for wheeled mobile robots. Int. J. Robotics Res. 34, 288–313. 10.1177/0278364914551773

[B20] Kim J. Alspach A. Yamane K. (2017). “Snapbot: a reconfigurable legged robot,” in 2017 IEEE/RSJ International Conference on Intelligent Robots and Systems (IROS), Vancouver, BC, Canada, September, 2017.

[B21] Klamt T. Behnke S. (2017). “Anytime hybrid driving-stepping locomotion planning,” in 2017 IEEE/RSJ International Conference on Intelligent Robots and Systems (IROS), Vancouver, BC, Canada, September, 2017, 4444–4451. 10.1109/IROS.2017.8206310

[B22] Liu D. Li C. Zhang J. Huang W. (2023). Robot service failure and recovery: literature review and future directions. Int. J. Adv. Robotic Syst. 20. 10.1177/17298806231191606

[B23] Machado J. A. T. Silva M. F. (2006). “An overview of legged robots,” in International symposium on mathematical methods in engineering, Cankaya, Ankara, Turkey, April, 2006.

[B24] Moreno A. Regina M. (2018). Fundamental study into rotor outwash and dust kick-up under mars-like conditions. NTRS.

[B25] Murata S. Kurokawa H. (2007). Self-reconfigurable robots. IEEE Robotics Automation Mag. 14, 71–78. 10.1109/MRA.2007.339607

[B26] Murphy R. (2000). Marsupial and shape-shifting robots for urban search and rescue. IEEE Intell. Syst. 15, 14–19. 10.1109/5254.850822

[B27] Ning M. Shao L. Chen F. Li M. Zhang C. Zhang Q. (2019). Modeling and analysis of a modular multilegged robot with improved fault tolerance and environmental adaptability. Math. Problems Eng. 2019, 1–17. 10.1155/2019/8261617

[B28] Noseworthy M. Brand I. Moses C. Castro S. Kaelbling L. Lozano-Perez T. (2021). “Active learning of abstract plan feasibility,” in Robotics: Science and Systems XVII (Robotics: Science and Systems Foundation), Virtually, July, 2021. 10.15607/RSS.2021.XVII.043

[B29] Pan T. Wells A. M. Shome R. Kavraki L. E. (2022). “Failure is an option: task and motion planning with failing executions,” in 2022 International Conference on Robotics and Automation (ICRA), Philadelphia, PA, USA, May, 2022, 1947–1953. 10.1109/ICRA46639.2022.9812273

[B30] Reid W. Göktoǧan A. H. Sukkarieh S. (2014). “Moving mammoth: stable motion for a reconfigurable wheel-on-leg rover,” in Australasian Conference on Robotics and Automation, ACRA, Melbourne, Australia, December, 2014.

[B31] Reid W. Perez-Grau F. J. Goktogan A. H. Sukkarieh S. (2016). “Actively articulated suspension for a wheel-on-leg rover operating on a Martian analog surface,” in Proceedings - IEEE International Conference on Robotics and Automation (IEEE), Stockholm, Sweden, May, 2016, 5596–5602. 10.1109/ICRA.2016.7487777

[B32] Salemi B. Moll M. Shen W. M. (2006). “SUPERBOT: a deployable, multi-functional, and modular self-reconfigurable robotic system,” in IEEE International Conference on Intelligent Robots and Systems, Beijing, China, October, 2006, 3636–3641. 10.1109/IROS.2006.281719

[B33] Showstack R. (2010). Mars rover enters new phase of mission. Eos, Trans. Am. Geophys. Union 91, 44. 10.1029/2010EO050003

[B34] Sreenivasan S. V. Dutta P. K. Waldron K. J. (1994). “The wheeled actively articulated vehicle (WAAV): an advanced off-road mobility concept,” in Advances in robot kinematics and computational geometry (Dordrecht, Netherlands: Springer Netherlands), 141–150. 10.1007/978-94-015-8348-0{_}14

[B35] Sreenivasan S. V. Waldron K. J. (1996). Displacement analysis of an actively articulated wheeled vehicle configuration with extensions to motion planning on uneven terrain. J. Mech. Des. 118, 312–317. 10.1115/1.2826886

[B36] Sreenivasan S. V. Wilcox B. H. (1994). Stability and traction control of an actively actuated micro???rover. J. Robotic Syst. 11, 487–502. 10.1002/rob.4620110604

[B37] Townsend J. A. Bellutta P. Keuneke M. Seibert M. Stroupe A. Wright J. (2014). “Mars exploration rovers 2004-2013: evolving operational tactics driven by aging robotic systems,” in SpaceOps 2014 Conference, Pasadena, CA, USA, May, 2014. 10.2514/6.2014-1884

[B38] Wilcox B. H. Litwin T. E. Biesiadecki J. J. Matthews J. B. Heverly M. C. Morrison J. C. (2007). ATHLETE: a cargo handling and manipulation robot for the moon. J. Field Robotics 24, 421–434. 10.1002/rob.20193

[B39] Yim M. Shen W. M. Salemi B. Rus D. Moll M. Lipson H. (2007). Modular self-reconfigurable robot systems Grand challenges of robotics. IEEE Robotics Automation Mag. 14, 43–52. 10.1109/MRA.2007.339623

